# Microsurgical Management of Craniocervical Dural Arteriovenous Fistula: A Case Report and Literature Review

**DOI:** 10.7759/cureus.65547

**Published:** 2024-07-27

**Authors:** Yoshio Araki, Made Bhuwana Putra, Tetsuya Tsukada, I Wayan Niryana, Ryuta Saito

**Affiliations:** 1 Neurosurgery, Japanese Red Cross Aichi Medical Centre Nagoya Daini Hospital, Nagoya, JPN; 2 Neurosurgery, Udayana University, Denpasar, IDN; 3 Neurosurgery, Nagoya University Hospital, Nagoya, JPN

**Keywords:** craniocervical junction, dural arteriovenous fistula, vascular malformation, arteriovenous shunt, arteriovenous malformation

## Abstract

Dural arteriovenous fistula (DAVF) of the craniocervical junction is exceptionally rare. The anatomy of the craniocervical junction area is very complex and is composed of the medulla and spinal cord along with intricate neurovascular structures. A thorough assessment of the angioarchitecture of the fistula is obligatory for choosing the most appropriate treatment for the patient. In this report, we describe the nuance of microsurgical obliteration of craniocervical junction DAVF utilizing intraoperative angiography.

A 38-year-old male in a normal state of health was referred to our hospital for an abnormality in his brain checkup. Workup diagnostics showed a DAVF on the craniocervical junction area with feeders from ascending pharyngeal, vertebral, and occipital arteries, with the draining vein mainly to the basal vein of Rosenthal. Microsurgical obliteration of the main draining vein was done with the help of intraoperative digital subtraction angiography with a good outcome.

Craniocervical DAVF is a rare entity. Meticulous evaluation of arterial and venous fistula points is necessary to decide the best treatment option for this case. Microsurgical obliteration is a feasible and more straightforward procedure for treating craniocervical DAVF.

## Introduction

Dural arteriovenous fistula (DAVF) of the craniocervical junction is exceptionally rare, comprising less than 2% of all DAVF [1]. The anatomy of the craniocervical junction is particularly intricate and incorporates the brainstem and spinal cord with upper spinal and lower cranial nerves along with various arteries and ligaments connecting the occipital bone to the axis and atlas [2]. Evaluation of the angiographic architecture in this type of DAVF is challenging, with convoluted and tortuous feeding arteries and various possibilities of draining veins. A thorough assessment of the involved vascular structure is necessary to determine the best treatment option, which can be either endovascular or direct microsurgical obliteration of the fistula [1]. In this report, we describe the nuance of assessing and managing the craniocervical junction DAVF.

## Case presentation

**Figure 1:**
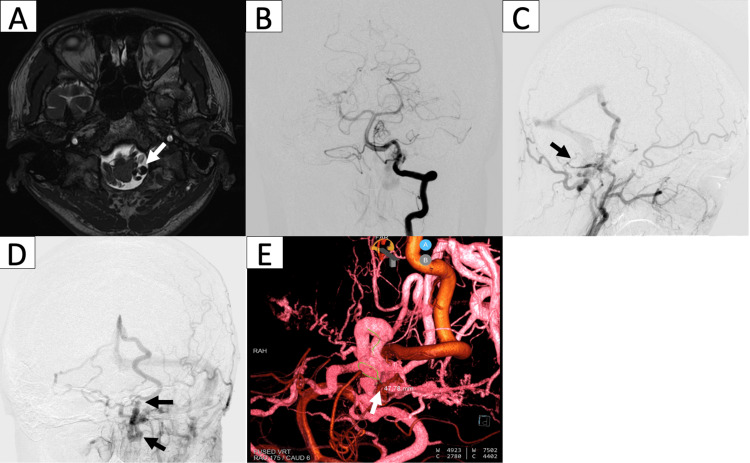
Preoperative imaging MRI shows a flow void (white arrow) on the T2-weighted image at the level of the foramen magnum (a). DSA with vertebral artery (b) and external carotid artery (c) injections show fistula with feeding arteries from branches of the ascending pharyngeal artery (white arrowhead), occipital artery (black arrow), and vertebral artery (white arrow). Venous phase (d) shows a draining vein that continues into the basal vein of Rosenthal (black arrow) and descending posterior spinal vein (black arrowhead). 3D DSA of both external and vertebral arteries depict the shunt point (white arrow) and feeder arteries (white arrowheads) (e). DSA: digital subtraction angiography

A 38-year-old male in a normal state of health was referred to our hospital for an abnormality in his brain checkup. On magnetic resonance imaging (MRI), we found an incidental finding of a flow void on a T2-weighted image at the level of the foramen magnum, presumably a vascular malformation. He had no complaints regarding headache, tinnitus, weakness, or any cranial nerve deficits. Further workup with digital subtraction angiography (DSA) was performed and showed multiple feeders from the hypoglossal and jugular branches of the ascending pharyngeal artery, mastoid branch of the occipital artery, and meningeal branch of the vertebral artery. These numerous feeders converged on the left lateral dura of the foramen magnum, where they formed a localized shunt point. The main draining vein originated from this shunt point, with one draining toward the basal vein of Rosenthal and the petrosal vein, and another descending into the posterior spinal vein of the spinal cord. We proceeded with direct microsurgical obliteration of the fistula in conjunction with intraoperative DSA in a hybrid operating theater. Figure 1 shows the preoperative imaging findings.

Surgical procedure

The patient was positioned supine, and femoral access was placed before turning the patient into a prone position. A linear midline incision followed by suboccipital craniotomy extending laterally to the left side was done. The cisterna magna was opened, allowing evacuation of the cerebrospinal fluid, which helps in slackening the cerebellum. Dissection of the lateral cerebellomedullary cistern revealed the shunt point with a tangled DAVF complex and a draining vein directed to the posterior spinal vein just next to the left vertebral artery. A clip was placed to obliterate the draining vein just adjacent to the shunt point, and indocyanine green (ICG) showed complete obstruction of the draining vein. Intraoperative DSA confirmed the obliteration of the main draining vein and the shunt point of the DAVF.

Postoperative period

**Figure 2:**
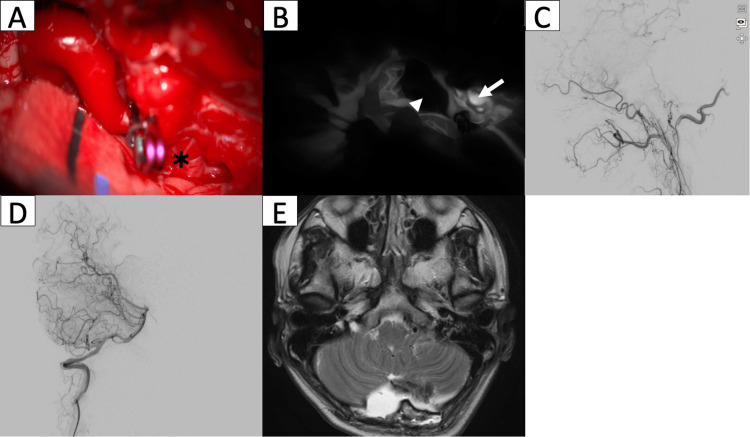
Intraoperative and postoperative imaging Intraoperative image shows a) suboccipital craniotomy with identification and clipping of the main draining vein just distal to the shunt point (asterisk). b) ICG image shows no residual flow on the main draining vein (white arrowhead) with residual flow at the arterial fistula (white arrow). Intraoperative DSA of the external carotid artery (c) and vertebral artery (d) along with postoperative MRI (e) confirmed complete obliteration of the main draining vein with no fistula remaining to be seen. ICG: indocyanine green; DSA: digital subtraction angiography

The patient tolerated the operation well, with uneventful complications during the postoperative period. No hemorrhage or ischemic complication was appreciated on follow-up head CT. No sign of new ischemia was found on the follow-up MRI either, and the fistula point was shown to have entirely disappeared along with the previously engorged draining vein. Figure 2 shows the intraoperative and postoperative imaging findings. The patient started early ambulation and was discharged on postoperative day 12. During his one-month follow-up, the patient was independent, with normal activities of daily living and no recurrent hemorrhage up to the last follow-up period.

## Discussion

Craniocervical junction DAVF represents a small fraction of DAVF situated at either the foramen magnum or craniocervical junction. These fistulas are considered non-sinus type and usually drain directly into pial/cortical veins. Craniocervical junction DAVF represents 2% of all DAVF in the central nervous system, with five times male predominance [1]. Cerebral venous thrombosis (CVT) was believed to be the main cause of DAVF development. CVT will cause venous hypertension, triggering hypoxia-induced angiogenesis or reopening the previously existing microchannel with subsequent fistulous connection formation [3]. Other authors proposed an inflammatory reaction to the emissary veins as one of the causes of the non-sinus type of DAVF development [3,4]. Contrary to the sinus drain, which will compensate for a more rapid flow, cortical venous drainage causes a substantial rise in hemodynamic stress on the vein. This retrograde flow will result in venous engorgement and varix development, which may rupture, causing subarachnoid hemorrhage (SAH) [1]. Further, the epidural fistula may cause a mass effect on the surrounding nerve or medulla, which may cause myelopathy [5]. Craniocervical DAVF may be a slow and progressive course in nature with insidious symptoms that may cause a delay in diagnosis [1]. Craniocervical junction DAVF may have a diverse and non-specific clinical presentation, including SAH, myelopathy, and lower cranial nerve palsy [1]. Some other symptoms may include cerebellar symptoms, radiculopathy, and occipital neuralgia [6]. Among those possible presentations, SAH is the most common, constituting around 37.5% of all craniocervical junction DAVF cases, followed by myelopathy [7].

The dural layer of the foramen magnum is supplied by several arterial branches. This includes the posterior and anterior meningeal branches of the vertebral artery, neuromeningeal branches of the ascending pharyngeal artery, and meningeal branches of the occipital artery. Neuromeningeal branches of the ascending pharyngeal artery course into the hypoglossal canal and jugular foramen while meningeal branches of the occipital artery may join the posterior meningeal artery to vascularize the posterior aspect of the posterior fossa [2]. Other possible vascular supplies are from the posterior inferior cerebellar artery, posterior auricular artery, and middle meningeal artery [1]. The majority of the craniocervical DAVF cases have unilateral vertebral artery feeders. Fistula with multiple feeders accounts for around 25% of all cases [8]. This is aligned with our case, as the feeder arteries to the shunt point were formed by the neuromeningeal (hypoglossal and jugular) branches of the ascending pharyngeal artery along with meningeal branches of both vertebral and occipital arteries. 

The draining vein of craniocervical junction DAVF can be categorized into two distinct types: the descending type, which drains into the perimedullary vein, and the ascending type, which drains into either the dural venous sinus or basal venous system. The ascending vein may eventually drain into the cavernous sinus, inferior petrosal sinus, or basal vein. Descending draining veins typically drain into the radiculomedullary or either the anterior or the posterior spinal vein of the spinal cord. The direction of venous drainage may be attributed to the presenting symptoms. Descending draining veins into the spinal or medullary vein may cause spinal cord edema, which manifests as myelopathy and tetraplegia if left untreated. Ascending intracranial drainage, especially into the cortical vein, was associated with the clinical presentation of SAH [9].

Currently, there is no distinct categorization for craniocervical junction DAVF. Based on the classic classification by Borden et al., our case was classified into Borden type 3 with direct pial venous drainage and early varix development. This type of arteriovenous fistula has a significantly high risk of rupture with an up to 10% annual hemorrhage rate and may increase to 21% as the venous varix develops. This may lead to a possible catastrophic event and should be considered for treatment [10]. Uda et al. mention the possibility of the development of pial arteriovenous malformation following the formation of DAVF, which has to be analyzed in scrutiny on the angiographic study before proceeding to the treatment [11].

Management of craniocervical junction DAVF can be either microsurgical or through an endovascular approach. Endovascular treatment may utilize either transvenous or trans-arterial approaches. The goal of the treatment is to completely occlude the shunt point, mainly the draining vein. A thorough angiographic evaluation to assess the feeding arteries, site of the shunt point, and draining vein must be done before the treatment decision is made [12]. In our case, the transvenous route was not feasible due to distant and tortuous venous access because of the non-sinus nature of the DAVF with tortuous veins. Arterial embolization of the ascending pharyngeal artery is considerably high risk because of the possibility of extravasation into the neuromeningeal branches, causing neurological deficits. Other feeding arteries from the mastoid branches of the occipital artery and the meningeal branches of the vertebral artery are particularly tortuous and thus not ideal for catheter access, not to mention the possibility of embolic agent extravasation into the neuromeningeal branches of the ascending pharyngeal artery. In the case of craniocervical junction DAVF, endovascular treatment is also associated with a higher risk of incomplete occlusion and recanalization after embolization [5,12]. Thus, microsurgical obliteration of the fistula was believed to be a more straightforward method and favored over the endovascular approach. In one series by Jeng et al., complete obliteration of the fistula was achieved in 75% of endovascular treatment compared to 98% from surgical management [13]. In their report, Hosseini et al. also suggested that although endovascular is a less invasive and efficient modality in treating DAVF, there is a higher risk of recanalization rates and residual fistula post-embolization than direct surgical management. Surgery may also offer direct visualization and neural decompression if necessary [14].

The surgical strategy in managing DAVF is to obliterate the main cortical draining vein with either coagulation or microsurgical clips [12]. Clip placement has to be as close as possible to the shunt point to prevent the risk of venous rupture proximal to the clip and prevent the possibility of recurrent fistula formation. While further dural fistula coagulation may be done, in our case, the proximity of the dural fistula to lower cranial nerves impedes the possibility of cauterization, preventing the risk of lower cranial nerve injury. With the obliteration of the main venous drainage, the low-flow arterial fistulous complex will naturally thrombose and disappear over time [15]. While the post-clipped flow of the draining vein can be evaluated through ICG, intraoperative DSA allows 3D angioarchitecture evaluation of the DAVF complex to ensure a complete obliteration of the fistula before closing the surgical site. Thus, if available, a hybrid operative theater should be utilized in the microsurgical management of DAVF. With complete obliteration of the fistula, the disease will cease to progress, along with the risk of hemorrhage [11]. If left untreated, severe disability may occur in 50% of the cases. In that case, despite successful surgery, clinical improvement may only be achieved by about two-thirds of the patients [1].

## Conclusions

Craniocervical DAVF is a particularly rare entity. Meticulous evaluation of arterial and venous fistula points is necessary to decide the best treatment option for this type of case. Microsurgical obliteration is a feasible and more straightforward procedure for treating craniocervical DAVF.
